# Light People: Prof. Juejun Hu, exploring the light

**DOI:** 10.1038/s41377-024-01583-2

**Published:** 2024-08-27

**Authors:** Tingting Sun

**Affiliations:** Light Publishing Group, Changchun Institute of Optics, Fine Mechanics and Physics, Chinese Academy of Sciences, No. 3888 Dong Nanhu Road, Changchun, 130033 China

**Keywords:** Integrated optics, Photonic devices

## Abstract

Professor Juejun Hu was admitted by Tsinghua University as top scorer in the science college entrance examination of Fujian Province. After graduating, he went to MIT to pursue further studies, where he continued to excel and became a faculty member. Each step of his journey has been marked by extraordinary achievements, setting a standard that few can match. Today, Prof. Hu is recognized as a leading expert in integrated photonics and optical materials. His pioneering research has not only advanced the frontiers of academia but also made significant impacts on industrial applications. In this interview, we invite you to delve into Prof. Hu’s research world, exploring his unique insights into technological innovation and how he uses the power of science to shape the future.

## Abstract (web summary)

Prof. Juejun Hu is recognized as a leading expert in integrated photonics and optical materials. His pioneering research has not only advanced the frontiers of academia but also made significant impacts on industrial applications.

## Graphical abstract

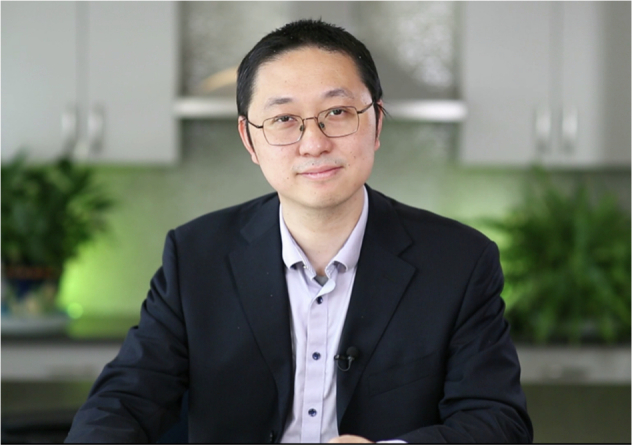

**Figure:**
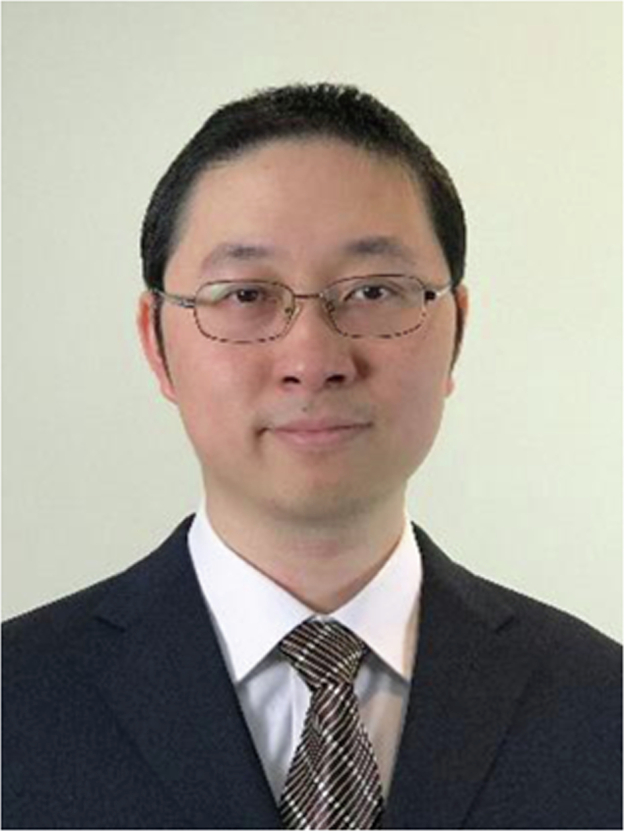
Prof. Juejun Hu

**Short Bio**

**Juejun Hu** is currently the John F. Elliott Professor of Materials Science and Engineering at MIT. Prior to joining MIT, he was an assistant professor at the University of Delaware. His research primarily focuses on integrated optics and photonics. Prof. Hu has authored and coauthored >150 refereed journal publications, and he has been recognized with the SPIE Early Career Achievement Award, the Robert L. Coble Award from the American Ceramic Society, the Vittorio Gottardi Prize from the International Commission on Glass, the NSF CAREER award, and the DARPA Young Faculty Award, among others. Hu is a fellow of Optica, SPIE, and the American Ceramic Society

**Q1: Could you please briefly introduce your current research focus?**

**A1:** Our group’s research focuses on two main directions: integrated photonics and metasurface optics. In integrated photonics, we are developing novel photonic packaging and on-chip spectroscopic sensing methods. We are also working on new heterogeneous integration technologies to seamlessly incorporate new materials into the standard foundry fabrication process flow, with the examples include nonvolatile phase change materials, magnetooptical/electro-optic crystals, non-traditional polymer and semiconductor materials, etc. On the metasurface optics front, our research covers their design theory and algorithm, nanofabrication, as well as device integration and packaging with other optoelectronic components for applications such as imaging, sensing, display, optical communications and LiDAR.

**Q2: Photonic Random-Access Memories (P-RAM) are the essential components for photonic computing, what are the shortcomings of traditional P-RAM? What are the advantages of the multistate electrically programmed low-loss nonvolatile photonic memory based on a broadband transparent phase-change material**^1^ **that you demonstrated? What technical problems did this research work solve?**

**A2:** Nonvolatile optical memory has long been a missing link in on-chip integrated photonics. Chalcogenide phase change materials (PCMs) with amorphous-crystalline transitions have been widely recognized as one of the most promising paths towards realizing nonvolatile optical functionalities. Traditional GeSbTe (GST) based PCMs are degenerate semiconductors in their crystalline phase, and strong free carrier absorption results. This high optical absorption leads to severe loss penalties in devices based on GST. Back in 2017 we first demonstrated a broadband transparent PCM, Ge-Sb-Se-Te (GSST)^2^, showing that a large refractive index contrast and bi-state transparency can be simultaneously achieved. In the following years we demonstrated a cohort of reconfigurable integrated photonic and metasurface devices based on these low-loss PCMs. The optical memory^1^ demonstrated through a collaboration with Prof. Volker Sorger’s group at University of Florida is a recent example. We realize 4-bit, electrically programmed on-chip photonic memories which can be stably cycled for well over 500,000 cycles. This represents a major improvement over prior state-of-the-art on reliability of on-chip PCM optical devices.

**Figure:**
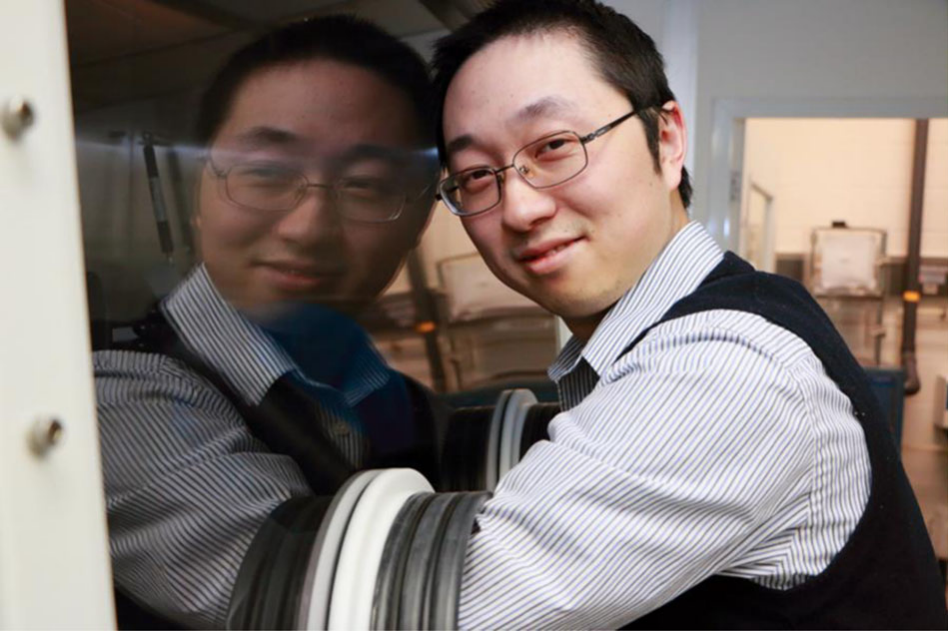
Prof. Hu in the lab

**Q3: Your team has developed a novel reconfigurable metasurface using low-loss optical phase-change material** ^3^ **, opening up new pathways for realizing various reconfigurable metasurface devices. Could you introduce the main research inspiration and ideas behind this work?**

**A3:** Compared to prior work on PCM-based reconfigurable metasurfaces, the main novelty of our work is the first demonstration of an electrically switched PCM metasurface. Electrical control can drastically simplify integration and packaging of these devices into functional modules, which is critical if they were to be deployed for practical applications. Meanwhile, using transparent PCMs also enable low-loss metasurfaces operating in a transmission mode rather than reflection, and this advantage has been validated in our more recent work^4^.

**Q4: Reconfigurable optical metasurfaces are rapidly emerging as a major frontier in photonics research, development and commercialization** ^5^ **. Could you introduce the latest advances in reconfigurable metasurface technology and applications? What opportunities and challenges are currently being faced?**

**A4:** In principle, reconfigurable optical metasurfaces can lead to light-weight, fast, and high-resolution spatial light modulators and filters that can potentially also operate at wavelengths difficult to access using traditional techniques (e.g., mid- or long-wave infrared). This is of significant importance to applications including imaging, analog optical computing, and optical beam forming/control. However, their overall performance still remains to be further improved before they can truly compete with traditional optics in practical applications. Further improving their performance, reliability and consistency, as well as realizing cost-effective and scalable manufacturing are necessary to enable their widespread adoption in industry.

**Figure:**
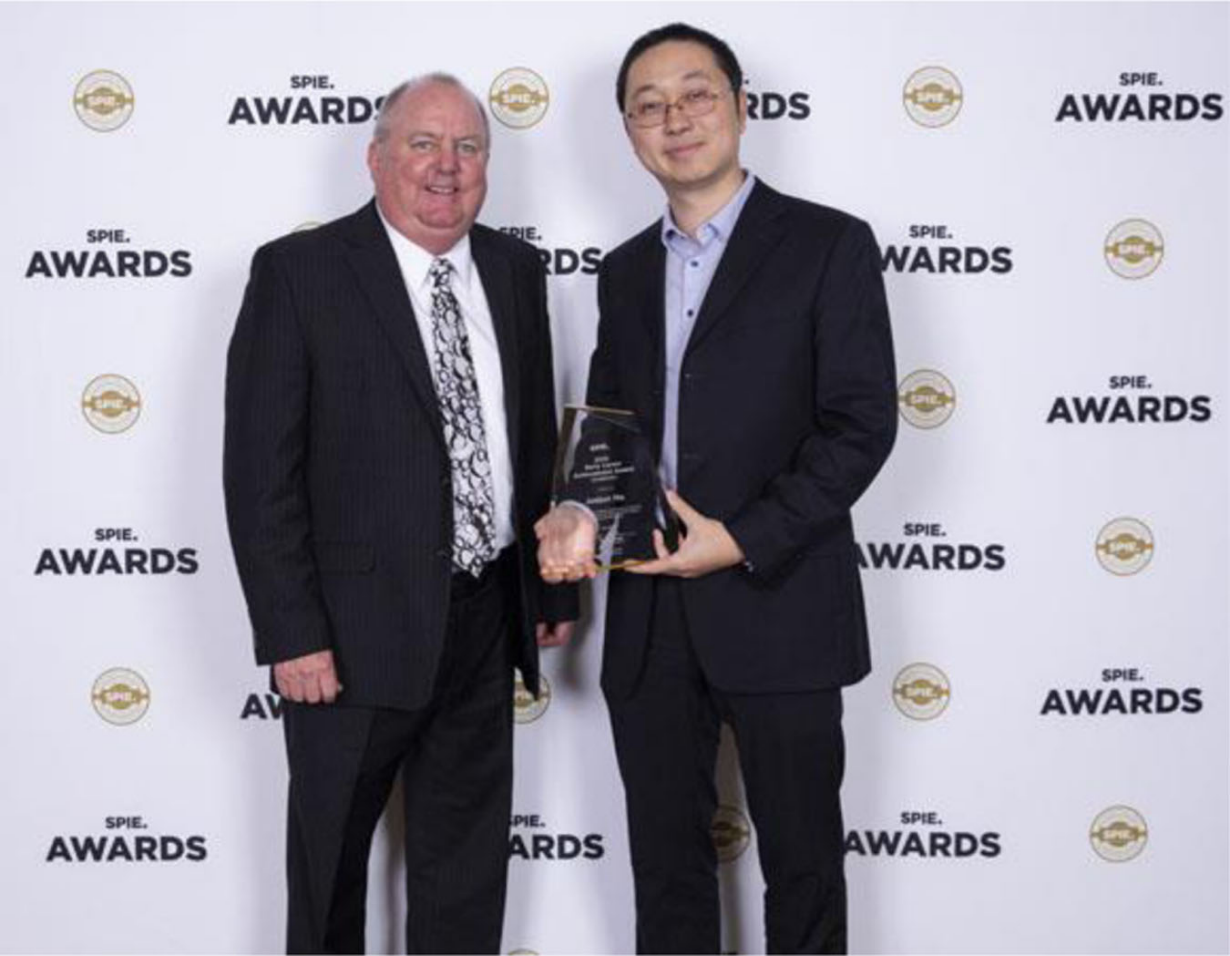
Prof. Hu being awarded the 2019 SPIE Early Career Achievement Award

**Q5: In recent years, wide-field-of-view metasurface optics has seen rapid development. Could you discuss about its current status and future trends? What are the emerging applications?**

**A5:** As we summarized in a recent review^6^, wide field-of-view metasurface optics have made major strides over the past few years. Several frontiers in this area include their integration with classical optical elements, expanding spectral bandwidth, exploring additional novel functionalities, and co-optimization with backend data processing algorithms. With unique and proven advantages such as large field-of-view, light-weight, ultra-compact footprint, and compatibility with wafer-scale manufacturing and packaging, metasurface optics are poised to make a significant impact on markets spanning consumer electronics, automotive sensing, robotics, and medical imaging.

**Q6: What are the key steps for wide-field-of-view Metalens to move towards commercialization?**

**A6:** Their commercial deployment is imminent over the next year or two, if not sooner. At 2Pi, we have demonstrated manufacturing of metasurface optics on 300 mm wafer platforms through partnering with a leading commercial foundry, and matured their integration into imaging and sensing modules. Please certainly do not hesitate to reach out to us if you see any applications that our technology may help with.

**Figure:**
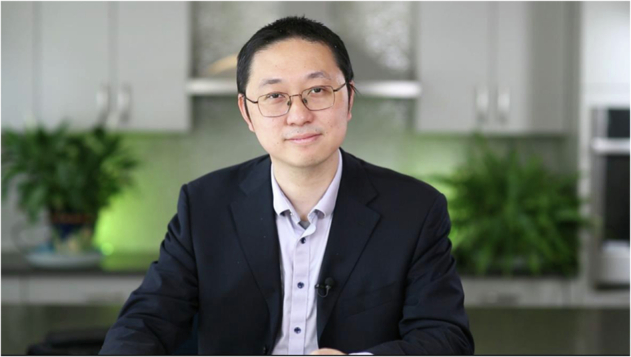
Teaching an online class on Integrated Photonics

**Q7: Photonic integrated circuits (PICs) have broad application prospects in various fields such as optical computing and optical sensing. What are the technical bottlenecks in the packaging of PICs? What are the innovations of the two-photon lithography for integrated photonic packaging**^7^ **that you proposed? What issues should be noted in the implementation process?**

**A7:** Photonic packaging nowadays relies on dedicated instruments for optical alignment and coupling to the photonic integrated circuit (PIC) chip. Coupled with the comparatively smaller market size of PICs compared to electronic IC chips, photonic packaging is yet to reach the economy of scale needed to drive down cost. Moreover, classical techniques for optical coupling exemplified by edge coupling and grating coupling have their respective drawbacks. Our method based on freeform micro-optical reflectors^8,9^ combines the best of both worlds, in particular the large spectral bandwidth and low loss of edge coupling, as well as a surface-normal coupling configuration commensurate with wafer-level testing and high-density I/O, which are characteristic of grating coupling. We also proposed a design approach that circumvents computationally intensive optimization of freeform structures, and instead enables simple deterministic design of these couplers utilizing the Fermat’s principle in optics. Couplers designed using this approach achieve a low coupling loss of 0.5 dB (for SiN waveguides) / 0.8 dB (for Si waveguides) at 1550 nm wavelength with a remarkable 1-dB bandwidth exceeding 300 nm (for SiN waveguides) / 180 nm (for Si waveguides). Furthermore, the couplers are compatible with standard foundry processed photonic waveguides with no customization (for example removal of top waveguide claddings) needed. This is an important feature that facilitates seamless integration of the couplers with industry-standard PICs.

**Q8: You have been deeply involved in the fields of integrated optics and photonics for many years, achieving numerous outstanding research results. Have any of your research findings been translated into practical applications, and what are the prospects?**

**A8:** A case-in-point is waveguide-enhanced Raman spectroscopy (WERS). The technology can lead to highly sensitive chip-scale Raman probes with dramatically reduced cost compared to their traditional counterparts—an important feature for single-use sensors in bioprocess monitoring. Our work theoretically proven the advantages of WERS and first experimentally realized fiber packaging of WERS chips^10,11^, which paves the path towards the technology’s practical deployment. One of my former students, Jerome Michon founded InSpek, an MIT spin-off startup commercializing the WERS technology. Under his leadership, InSpek has matured the technology over the past few years and have successfully implemented it to real-time monitoring of industry-scale biosynthetic processes, significantly improving the product yield for their customers.

**Figure:**
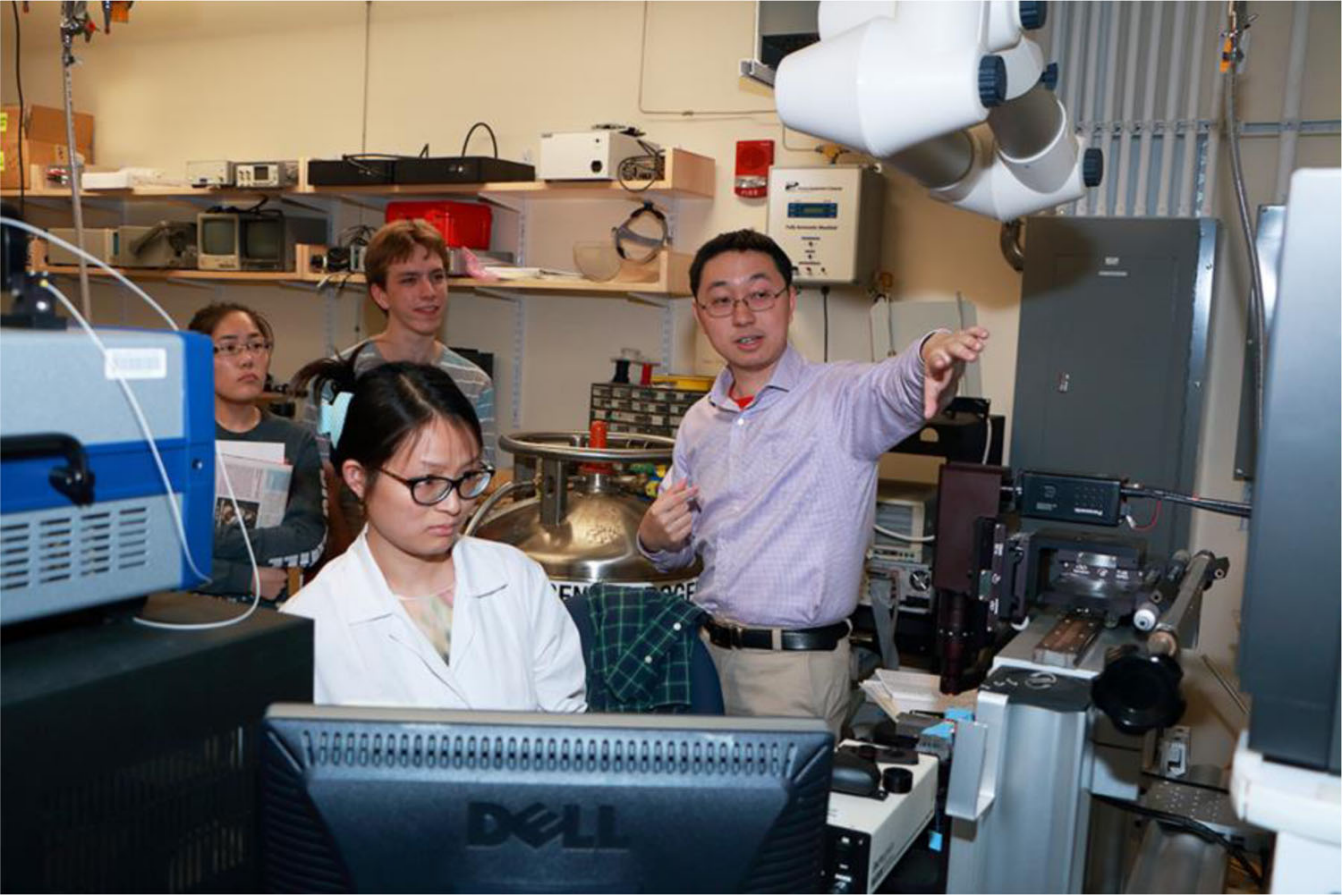
Prof. Hu in an outreach event explaining his research to visiting undergraduate students

**Q9: As a co-founder of 3 emerging photonics technology companies, what do you consider the key differences between conducting research and building an industry?**

**A9:** The aims are different. Research is gauged by intellectual merit (novelty and ingenuity of concepts and the potential to advance knowledge) and broader impacts (the potential to produce results relevant to practical applications that benefit the society). Commercialization is market and customer centric with the goal of realizing products that generate revenue and profit.

**Q10: How did you overcome the challenges you encountered in your research career?**

**A10:** This is a question about perseverance, and ultimately, motivation. There are tangible (and often short-lived) goals that one can work towards, such as getting a paper accepted, having a grant proposal awarded, receiving an award or a fellowship, and such. However, I believe that the only enduring objective that empowers one to forge forward overcoming all challenges must reside in the long-lasting impact of one’s work on the human race. Many of us who choose to pursue a research career have childhood dreams of becoming a scientist or engineer, where we get to witness exciting discoveries unfolding in front of our own eyes or invent ingenious gadgets nobody has conceived before that make people’s lives better. These innate dreams still live in me, and inspire myself to get a little step closer to the ideal every day.

**Figure:**
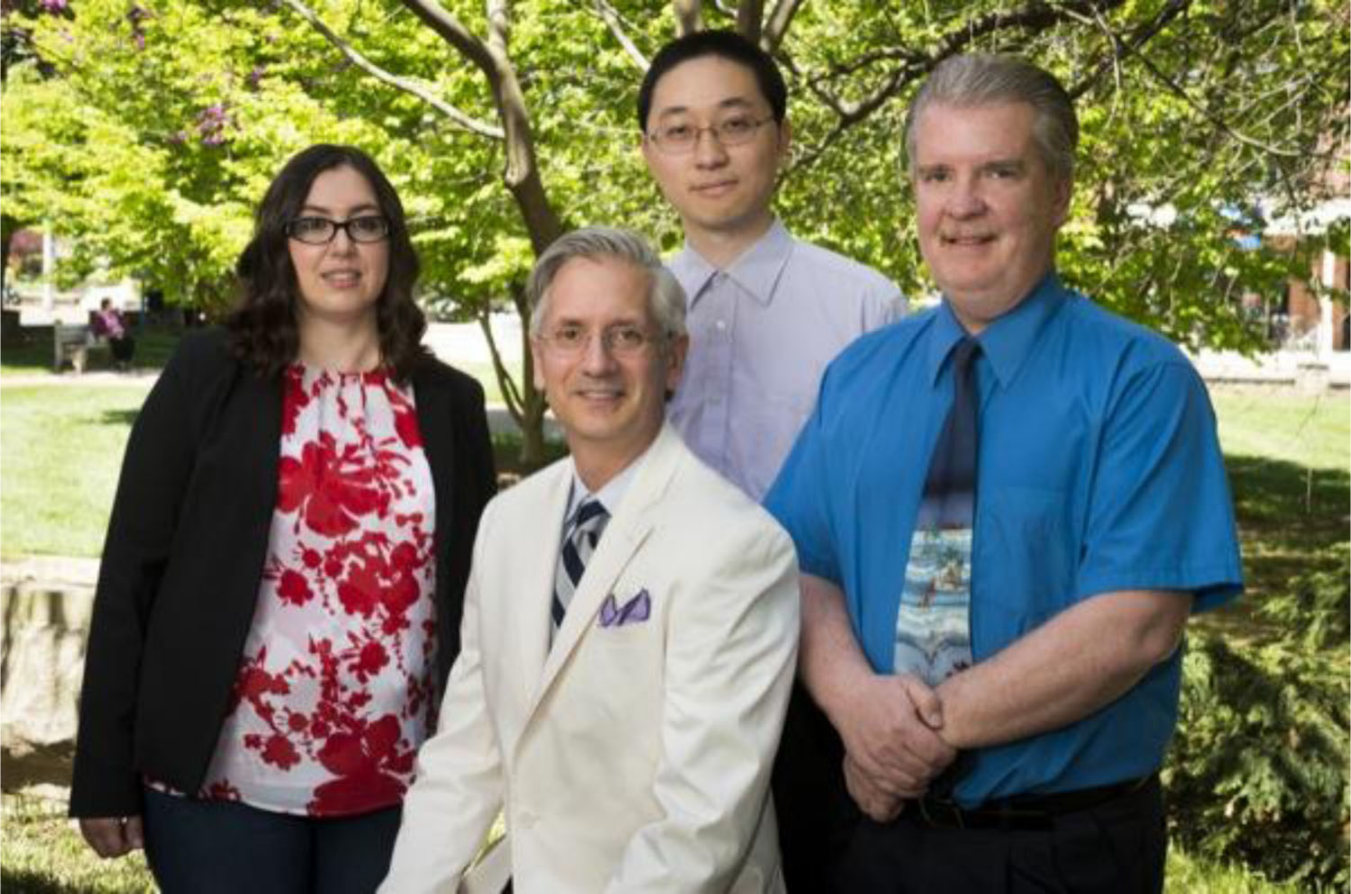
Prof. Hu winning the Excellence in Teaching Award at the University of Delaware

**Q11: MIT is a world-renowned top-tier institution and a dream destination for many young scholars. Why did you choose MIT? What was the experience of conducting research at MIT like?**

**A11:** I chose MIT? I think I was just incredibly lucky to get in!

I was excited and lucky to work with doctoral students and postdocs who were more excellent than myself—without them, I would have achieved nothing. It has been nearly 10 years since I became an assistant professor at MIT to now, I have had the privilege of working with 29 very good doctoral students and postdocs. They are the real heroes who venture into areas that no one else (of course included myself) stepped into, expand the horizon for our kind, and transform innovative ideas into reality (and in several cases, products). People sometimes comment that when one graduates/leaves a research group, s/he should be better than her/his advisor. They truly accomplished it. The intellectually stimulating discussions I had with them are among the most cherished memories I have throughout my time at MIT.

**Q12: MIT and Light: Science & Applications (Light) are collaborating to launch the MIT-Light Special Issue, and we greatly appreciate your acceptance to serve as the guest editor-in-chief for this special issue. What are your expectations for this special issue? Do you have any suggestions for the development of the Light journal?**

**A12:** We all witnessed the rapid expansion of the Light journal over the past few years, and it has been widely recognized as one of the top journals in the fields of optics. Many of the groundbreaking works in my field were published in Light, which attests to the recognition by the global optics and photonics community. Through this joint effort with Light, I am very impressed by the professionalism and efficiency of its editorial team, and we are gaining increasing confidence in Light’s continuing growth. I also hope that this special issue will become a venue for first-rate seminal work and further contribute to the development of the journal.

**Figure:**
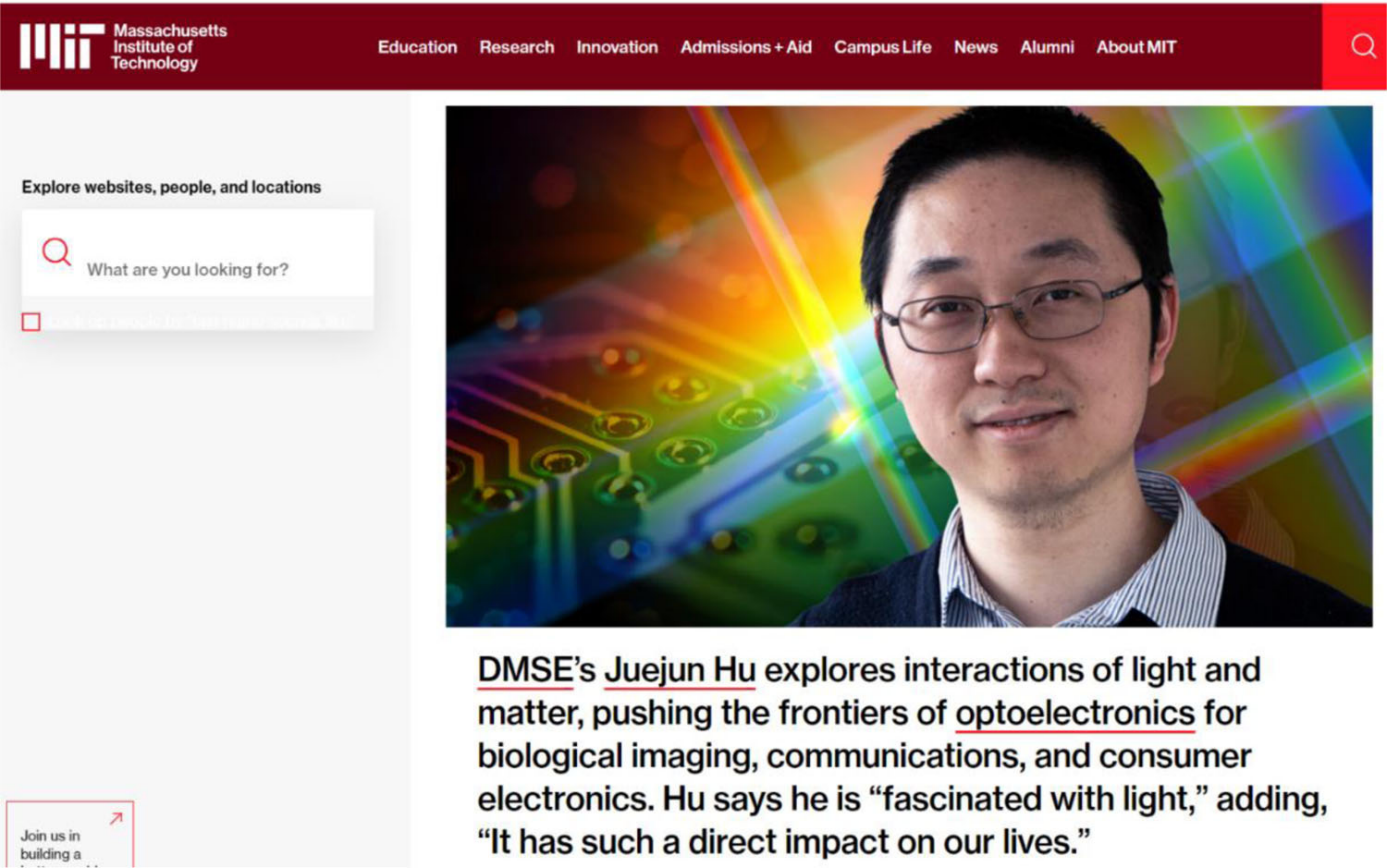
Prof. Hu spotlighted on MIT’s Homepage
